# Diseases of the Tongue

**Published:** 1885-12

**Authors:** 


**Diseases of the Tongue.** By HENRY T. BUTLIN, F. R. C. S., Surgeon and Demonstrator of Practical Surgery and Diseases of the Larynx, St. Bartholomew's Hospital, London. Illustrated with chromo-lithographs and engravings. Philadelphia: Lea Brothers & Co. 1885.

This is one of the series of clinical manuals for practitioners and students issued by the Leas. We have frequently had occasion to commend the volumes of the series. The present one is really a fairly complete treatise on the subject, and brings together much which would otherwise have to be laboriously sought for in the larger works. The plates which illustrate the work are admirable, and the book is worth more than the price for them alone. The author has clearly had a large practical experience, and writes well.

